# A bovine miRNA, bta‐miR‐154c, withstands *in vitro* human digestion but does not affect cell viability of colorectal human cell lines after transfection

**DOI:** 10.1002/2211-5463.13402

**Published:** 2022-03-31

**Authors:** Myrtani Pieri, Elena Theori, Harsh Dweep, Myrofora Flourentzou, Foteini Kalampalika, Maria‐Arsenia Maniori, Gregory Papagregoriou, Christos Papaneophytou, Kyriacos Felekkis

**Affiliations:** ^1^ 121343 Department of Life and Health Sciences University of Nicosia Cyprus; ^2^ The Wistar Institute Philadelphia Pennsylvania USA; ^3^ 54557 Molecular Medicine Research Center University of Cyprus Nicosia Cyprus

**Keywords:** bovine, colorectal cancer, digestion, epithelial cell lines, miroRNAs, XenomiRs

## Abstract

Colorectal cancer (CRC) is the third most frequent human cancer with over 1.3 million new cases globally. CRC is a complex disease caused by interactions between genetic and environmental factors; in particular, high consumption of red meat, including beef, is considered a risk factor for CRC initiation and progression. Recent data demonstrate that exogenous microRNAs (miRNAs) entering the body via ingestion could pose an effect on the consumer. In this study, we focused on bovine miRNAs that do not share a seed sequence with humans and mice. We identified bta‐miR‐154c, a bovine miRNA found in edible parts of beef and predicted via cross‐species bioinformatic analysis to affect cancer‐related pathways in human cells. When bovine tissue was subjected to cooking and a simulation of human digestion, bta‐miR‐154c was still detected after all procedures, albeit at reduced concentrations. However, lipofection of bta‐miR‐154c in three different colorectal human cell lines did not affect their viability as evaluated at various time points and concentrations. These data indicate that bta‐miR‐154c (a) may affect cancer‐related pathways in human cells, (b) can withstand digestion and be detected after all stages of an *in vitro* digestion protocol, but (c) it does not appear to alter epithelial cell viability after entering human enterocytes, even at supraphysiological amounts. Further experiments will elucidate whether bta‐miR‐154c exerts a different functional effect on the human gut epithelium, which may cause it to contribute to CRC progression through its consumption.

## Graphical abstract

MicroRNAs (miRNAs) are small RNA molecules present in foodstuffs. Here, we show that bovine miRNA, bta‐miR‐154c, is predicted to affect cancer‐related pathways in humans and can withstand a model of human digestion. However, it does not affect cell viability after uptake by epithelial cancer cells. Future studies will determine whether ingested miRNAs affect human enterocytes, thereby contributing to the progression of colorectal cancer.

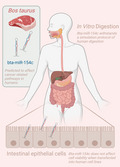

**Abbreviations**
CRC: colorectal cancer
GOBP: gene ontology biological process
miRNAs: microRNAs
MTT: 3‐(4,5‐dimethylthiazol‐2‐Yl)‐2,5‐diphenyltetrazolium bromide

CRC development is a multistep process in which the normal epithelium undergoes malignant transformation to a fully developed tumor through accumulations of genetic and epigenetic alterations [1]. Daily lifestyle, especially the western‐life diet is regarded as a possible causal factor for CRC where an increased risk has been consistently reported for long‐time consumption of red meat [2, 3]. Mechanistically, this dietary link has frequently been linked to the heme iron molecule of red meat [4] or attributed to chemical carcinogens arising during the cooking process [5], but many unknowns remain on the molecular mechanisms behind the strong epidemiological link between red meat and CRC. MicroRNA deregulation is another factor that has been consistently reported in CRC with altered expression miRNA patterns being associated with diagnosis, prognosis, and therapeutic outcomes of CRC [6].

Besides the endogenous microRNA deregulation observed in CRC, there is an interesting debate in recent literature on exogenous microRNAs found in edible sources and whether these could be transferred via the oral route, withstand digestion, and even regulate expression of human genes in a “cross‐species” manner [7, 8, 9, 10]. Some reports challenge these findings [11, 12, 13, 14, 15], whereas a number of other studies confirmed that, in contrast to the general conviction, some exogenous miRNAs appear to be absorbed in biologically meaningful amounts from nutritionally relevant doses from plants [16] and animals [17, 18] and affect gene expression in various tissues in humans [19]. Even though evidence of transfer of miRNAs from the diet to the blood is still inconclusive, some miRNAs have been shown to be resistant during digestion and to reach the enterocytes [20]. In the case of CRC initiation and progression, where the enterocyte shifts from the physiological to the malignant state, dietary miRNAs could exert their deregulating function without the need to cross the gut epithelium and be absorbed into the bloodstream, which makes the exogenous miRNA effect more plausible. Also, miRNAs are widely present in various foods and consist a basic component of our diet. Animal edible tissue, in particular, including bovine tissue, is enriched in microRNAs that have been shown to remain relatively stable irrespectively of food processing [3, 21]. However, there are very few studies examining the effect of miRNAs from meat and other animal products as compared to plant studies, due to the fact that animal miRNAs are practically identical to human ones making their detection and quantification more difficult [20]. Since red meat has been linked to CRC and in light of data depicting ingested miRNAs could pose an effect on the consumer, this study focused on the putative cross‐species effect of bovine miRNAs on the human enterocyte. To achieve this, we focused on bovine miRNAs that do not share their seed region sequence with humans. We also excluded miRNAs that share their seed sequence with mouse miRNAs in order to be able to perform future *in vivo* experiments.

Therefore, the scope of this study was three‐fold: (a) to identify bovine miRNAs found specifically in edible parts of beef that do not share a sequence with human or mouse miRNAs, (b) to examine whether any of these sequences are predicted by cross‐species bioinformatic analysis to affect human mRNAs and cancer‐related pathways, and (c) to select a candidate bovine miRNA and examine in proof‐of‐principle experiments whether it can withstand a human *in vitro* digestion protocol and affect the viability of human epithelial cell lines after transfection.

## Materials and methods

### Bioinformatic analysis

**Fig. 1:**
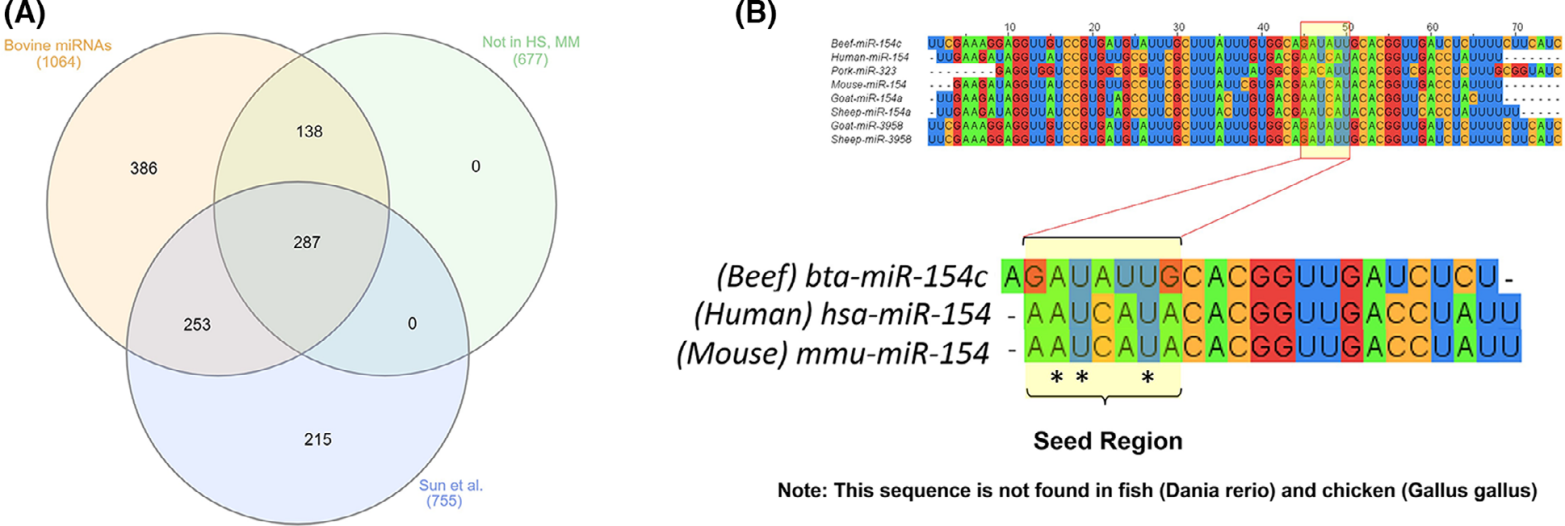
Bta‐miR‐154c is predicted to affect human genes involved in tumorigenesis. (A) Venn diagram depicting the filtering process of bovine miRNA selection. From a total of 1064 bovine miRNAs deposited in release 22.1 of miRBase (http://www.mirbase.org), we filtered for miRNAs that are highly expressed in edible parts of beef based on a literature search (Sun et al.) and do not share seed regions with miRNAs in humans or mice. We identified 287 bovine miRNAs fitting the above criteria. (B) Bta‐miR‐154c is the most highly abundant miRNA in bovine fat. Its seed region is not conserved between bovine, human, and mouse. It is also not conserved in pork but can be found in goat and sheep. This miRNA is not found in fish (*Danio* *rerio*) and chicken (*Gallus* *gallus*).

From a list of 1064 bovine miRNAs for *Bos taurus* found in miRBase (miRBase, release 22.1), we filtered for miRNAs that do not share common seed sequences with humans and mice. We also searched the literature for papers that report bovine miRNAs present in edible parts of beef and made their data available. We identified a paper from Sun et al, 2014 that explored the backfat miRNAome differences between cattle’s different developmental stages [22]. From a total of 287 miRNAs that supported both criteria as shown in the Venn diagram (Fig. 1A), we selected bta‐miR‐154c that was the top of the list miRNA presenting in the highest abundance in bovine adult fat (Table S1). We then evaluated the ability of this miRNA to target and regulate human genes, pathways, and diseases in a cross‐species bioinformatic analysis. This was achieved by collecting the experimentally verified and the putative target genes from miRTarBase for bta‐miR‐154c. The collated targets information was then mapped against the gene sets of KEGG pathways and gene ontology biological processes (GOBP) with the help of R‐statistical environment and with DAVID database. We also identified putative human mRNA targets for bta‐miR‐154c using the mirwalk2.0 software (http://zmf.umm.uni‐heidelberg.de/apps/zmf/mirwalk/). In order to identify human mRNA targets, we downloaded all human mRNA sequences from the RefSeq database. All the downloaded sequences were then scanned for possible binding sites of bta‐miR‐154c using miRWalk2.0, TagetScan, miRanda, and RNAhybrid algorithms. These 3 algorithms were locally executed to identify possible interactions among human genes and bta‐miR‐154c miRNA. Thereafter, we carried out pathways and GOBPs enrichment analyses to identify the regulation of bta‐miR‐154c on the human genome. This analysis resulted in a list of all the predicted human mRNAs, pathways and diseases predicted to be affected by the bovine bta‐miR‐154c. We continued with proof‐of‐principle *in vitro* experiments.

### *In vitro* human digestion protocol

In order to examine whether bta‐miR‐154c withstands cooking and human digestion, beef was purchased from the local butcher (Cypriot cattle population: *Bos taurus indicus*), bovine adipose and muscle tissues were separated and part of the tissue was cooked (200 °C, 45 min) and homogenized, and 5mg of each tissue was subjected to an *in vitro* digestion protocol of static human digestion as recommended and described in the international consensus paper by Minekus et al. 2014 [23].

### RNA Extraction and capillary electrophoresis

RNAs were extracted from the samples using the NucleoSpin RNA Kit (Macherey Nagel, UK), following a protocol for small RNA purification. Extracted RNA samples were analyzed on an Agilent 2100 Bioanalyzer using the agilent 2100 Expert Software in conjunction with the Bio‐SmallChip, which is especially indicated for small RNAs (length < 200 nucleotides). RNA integrity and quantification were assessed by performing a smear analysis of the electropherograms obtained by the capillary electrophoresis.

### Quantitative Real‐Time PCR

To quantify bta‐miR‐154c expression after cooking and digestion, we performed qRT‐PCR absolute quantification analysis. To this end, different known concentrations of the bta‐miR‐154c mimic (provided by Qiagen, Mature sequence (guide strand): 5’‐CUUUUUGCGGUCUGGGCUUGC‐3’, Cat. No: MSY0000242) were used. RNA from each sample was reverse‐transcribed using the miScript II RT Kit (Qiagen, UK) according to the manufacturer’s instructions. The sequence‐specific forward primer for mature bta‐miR‐154c was provided by Qiagen (miScript Primer Assay Qiagen, UK, Catalog number: MS00050575). Quantitative real‐time PCR was performed using the miScript SYBR Green PCR kit (Qiagen, UK) with the Universal miR qPCR Primer included in the kit. The reactions were performed on a LightCycler® (Roche Diagnostics, USA). The PCR conditions were 30 s at 95 °C, followed by 40 cycles at 95 °C for 5 s and 60 °C for 20 s. The logarithms of the different bta‐miR‐154c concentrations (x‐axis) were plotted against the Cp value (Y‐axis). The regression equation of the standard curve of the bta‐miR‐154c mimic was y = −3.5249x + 29.446, *R*² = 0.9985. Absolute quantification was determined for both bovine muscle and adipose tissue before and after cooking and digestion. Experiments were repeated 5 times and are depicted as mean ± SD, **P < 0.01.

### Sequencing of PCR products

To further examine and confirm that the PCR signals were indeed from bta‐miR‐154c, the PCR products were cloned into a vector and sequenced. Specifically, RNAs extracted either from the undigested raw bovine fat tissue (URF) or from the cooked bovine fat tissue after all stages of digestion (ICF) were reverse‐transcribed using the miScript II RT Kit (Qiagen, UK). cDNAs were added as templates in a standard PCR reaction using the Tag polymerase (Invitrogen, UK) and the specific forward primer to the bta‐miR‐154c miRNA (Qiagen, UK). The bta‐miR‐154 mimic was also used as a positive control in the PCR reaction. Resulting bands were gel‐purified and cloned into the pGEM‐T vector (Promega, UK) using the T4 ligase (Promega, UK). Plasmids were sequenced using BigDye V3.1 chemistry on an AB3130xl sequencer (Applied Biosystems, USA) and using standard T7 and SP6 primers.

### Cell culture

Caco‐2, SW620, and SW480 cells (All purchased from ATCC) were maintained using McCoy’s 5A medium (Sigma, St. Louis, USA) containing 10% horse serum (Biosera, France) and 100 IU·mL^−1^ of penicillin, and 100 mg·mL^−1^ of streptomycin (Sigma, UK) at 37 °C in a humidified atmosphere containing 5% CO_2_. Horse serum was used in all experiments (as opposed to standard FBS that originates from bovine tissue and could interfere with the current experimental approach). Cells were transfected with bta‐miR‐154c mimic at two concentrations (25 nm and 100 nm) using Lipofectamine 2000 (Invitrogen, UK).

### Relative quantification qRT‐PCR

To quantitate bta‐miR‐154c expression after transfection at various time points, total RNA was extracted from cells using the Nucleospin miRNA kit (Macherey Nagel, UK). The isolated total RNA was reverse‐transcribed using the miScript II RT Kit (Qiagen, UK) according to the manufacturer’s instructions. Relative expression was calculated via the comparative cycle threshold (Ct) method using the expression of U6 small nuclear RNA (RNU6) as reference. The sequence‐specific forward primer for mature bta‐miR‐154c and RNU6 internal control were provided by Qiagen (miScript Primer Assay, Qiagen, UK) with Catalog numbers: MS00050575 and MS00033740, respectively. Quantitative real‐time PCR was performed using the miScript SYBR Green PCR kit (Qiagen, UK) with the Universal miR qPCR Primer included in the kit. The reactions were performed on a LightCycler® (Roche Diagnostics, USA). The PCR conditions were 30 s at 95 °C, followed by 40 cycles at 95 °C for 5 s and 60 °C for 20 s. The 2‐▵Ct(2‐[(Ct of gene) ‐(Ct of reference)]) method was used for analysis.

### MTT cell viability assay

The 3‐(4,5‐dimethylthiazol‐2‐yl)‐2,5‐diphenyltetrazolium bromide (MTT) assay was performed for the determination of cell viability of the cell lines tested. Cells were seeded at 0.5 × 10^4^ cells·well^−1^ in a 96‐well plate followed by incubation at 37 °C for 24 h, then transfected using lipofectamine 2000 (Invitrogen, UK) with bta‐miR‐154c mimic at a low and a high concentration (25 nm and 100 nm). The control group was treated with a scramble mimic sequence. This negative control is an RNA sequence similar to the mature mimic sequence but with no known targets in human mRNAs as provided by Qiagen (catalog number: SI03650318). Negative control (scramble) mimic was used at the highest concentration (i.e., 100 nm) in order to make it as “stringent” control as possible. A second control consisted of transfection reagent only without any RNA (lipo only condition). After incubation for 24, 48, 72, and 96 h, 0.5 mg·mL^−1^ MTT solution was added to each well and the plate was further incubated at 37 °C. The supernatant was removed, and DMSO was added to each well to solubilize the purple formazan crystals. The absorbance was measured at 570 nm using a microplate reader (Victor 2030, Perkin Elmer). Data are the means of six measurements of OD, with experiments repeated ≥three times. Data are presented as mean ± SD, n.s. represents not significant.

### Statistical analysis

All statistical analyses were performed using graphpad Prism version 9.3.1 for Windows, GraphPad Software, La Jolla California USA, www.graphpad.comPrism. All data are expressed as mean ± standard deviation (SD). The ANOVA and *post hoc* Tukey’s tests were used to compare different samples, and a *P* value < 0.05 was considered significant.

## Results

### Selection of bta‐miR‐154c, a bovine microRNA, predicted to target cancer‐related pathways in human cells

As shown in the Venn diagram produced by the InteractiVenn web‐based tool [24] in Fig. 1A, from a total of 1064 bovine miRNAs deposited in release 22.1 of miRBase, we filtered for miRNAs that are highly expressed in edible parts of beef based on a literature search [22] and do not share seed regions with miRNAs in humans or mice. Our analysis resulted in 287 bovine miRNAs that fit the above criteria (Fig. 1A). We then sorted those miRNAs based on their abundance in bovine tissue and selected the top of the list miRNA, bta‐miR‐154c, for further analysis. Sequence alignment analysis showed that bta‐miR‐154c shares a seed sequence with other miRNAs found in red meats such as goat and sheep but not in fish or poultry (Fig. 1B).

We then hypothesized that this miRNA expressed in beef but not in humans could be absorbed by the colonic epithelium and affect human genes involved in cancer initiation or progression. Bta‐miR‐154c was predicted via cross‐species bioinformatic analysis to affect a high number of human genes based on 5 different algorithms (Table 1 and Table s1), and to affect cancer‐related pathways (Table 2 and Table S2) and diseases (Table S3) in humans.

**Table 1:** Top 20 human genes targeted by bta‐miR‐154c. Cross‐species bioinformatic analysis using 5 different algorithms showing the predicted human genes that are targeted by bta‐miR‐154c.

Gene	EntrezID	miRWalk	miRanda	RNAhybrid	Targetscan	PITA	Predicted by # programs
ACTN2	88	1	1	1	1	1	5
ADH1B	125	1	1	1	1	1	5
ALDOB	229	1	1	1	1	1	5
AMBN	258	1	1	1	1	1	5
ANXA7	310	1	1	1	1	1	5
FAS	355	1	1	1	1	1	5
AXL	558	1	1	1	1	1	5
BAI3	577	1	1	1	1	1	5
BBS2	583	1	1	1	1	1	5
BMP5	653	1	1	1	1	1	5
BMPR1A	657	1	1	1	1	1	5
DST	667	1	1	1	1	1	5
ZFP36L1	677	1	1	1	1	1	5
CA8	767	1	1	1	1	1	5
CACNA1D	776	1	1	1	1	1	5
CD8A	925	1	1	1	1	1	5
CD28	940	1	1	1	1	1	5
ENTPD5	957	1	1	1	1	1	5
CDC42	998	1	1	1	1	1	5
CHRM1	1128	1	1	1	1	1	5

**Table 2:** Top 20 enriched pathways targeted by bta‐miR‐154c. Cross‐species bioinformatic analysis showing the cancer‐related pathways that are targeted by bta‐miR‐154c.

Pathway	Name	n sig in PW	n nonsig in PW	*P* value
hsa04723	Retrograde endocannabinoid signaling	60	43	1.34E‐07
hsa05212	Pancreatic cancer	42	24	3.49E‐07
hsa05032	Morphine addiction	54	39	6.34E‐07
hsa04713	Circadian entrainment	55	42	1.42E‐06
hsa04068	FoxO signaling pathway	70	63	2.49E‐06
hsa05200	Pathways in cancer	148	179	3.08E‐06
hsa04724	Glutamatergic synapse	60	56	2.47E‐05
hsa04727	GABAergic synapse	49	41	2.31E‐05
hsa04151	PI3K‐Akt signaling pathway	151	195	2.64E‐05
hsa04919	Thyroid hormone signaling pathway	60	59	6.51E‐05
hsa04020	Calcium signaling pathway	84	97	0.00012657
hsa04150	mTOR signaling pathway	34	26	0.000139044
hsa04725	Cholinergic synapse	56	57	0.000200041
hsa04360	Axon guidance	61	66	0.000319107
hsa05210	Colorectal cancer	34	28	0.000325117
hsa05205	Proteoglycans in cancer	99	126	0.000392198
hsa04390	Hippo signaling pathway	71	83	0.000501011
hsa04144	Endocytosis	90	113	0.000514809
hsa04261	Adrenergic signaling in cardiomyocytes	69	80	0.000510429
hsa04510	Focal adhesion	91	115	0.000556128

### Bta‐miR‐154c in bovine tissue can be detected intact after subjected to a static *in vitro* system of human digestion

In order to examine whether bta‐miR‐154c withstands cooking and human digestion, bovine adipose and muscle tissues were separated, and part of the tissue was cooked, homogenized and subjected to an *in vitro* digestion protocol of static human digestion. Small RNAs including microRNAs were extracted after each step of the digestion protocol and integrity, and quantification was assessed.

**Fig. 2:**
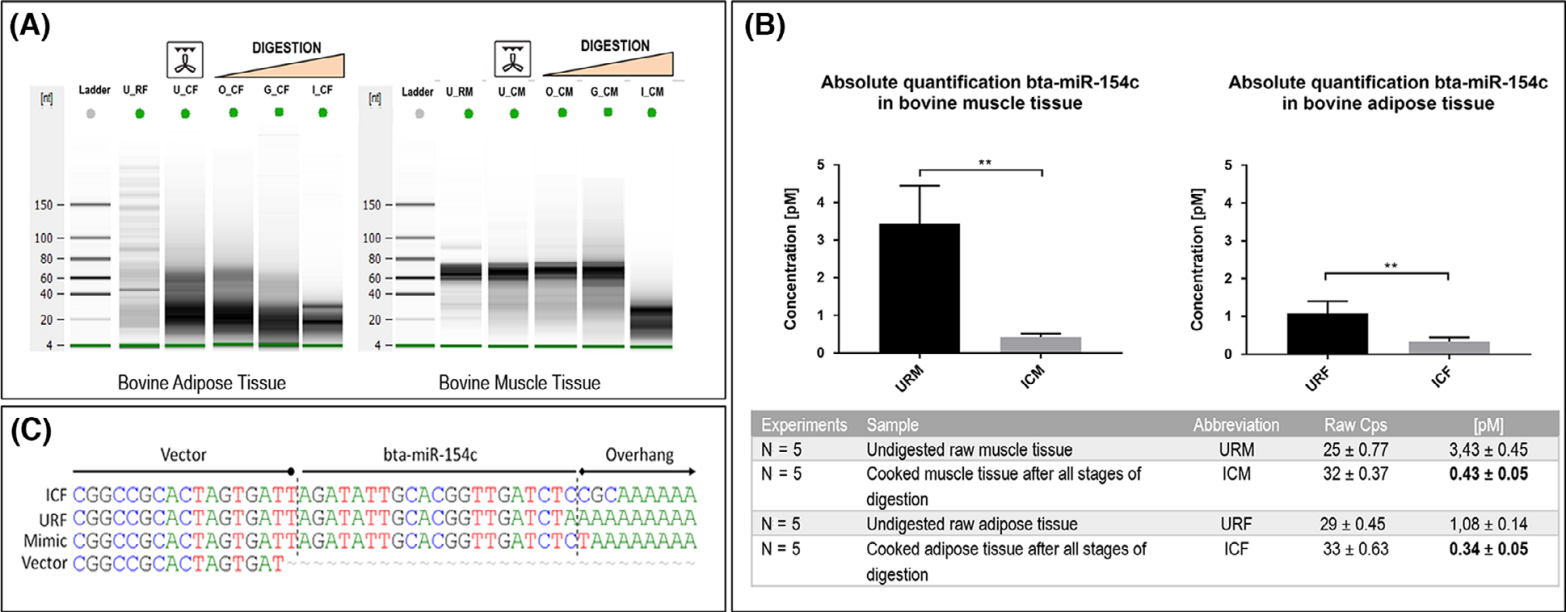
Bta‐miR‐154c withstands cooking and digestion. (A) In order to determine the bta‐miR‐154c presence during the digestion course, individual 30‐mg samples were collected for total RNA extraction after each step of digestion (oral, gastric, and intestinal) and RNAs were analyzed on an Agilent 2100 Bioanalyser. The ladder represents RNA fragments with molecule weights measured in kilo‐Daltons (kDa). Figure depicts an electronic representation of the capillary electrophoresis gels from small RNAs extracted by the adipose tissue (left) and the muscle tissue (right) after cooking and after the various steps of digestion. Samples are coded as: U_RF (undigested raw fat), U_CF (undigested cooked fat), O_CF (After oral phase of digestion, cooked fat), G_CF (After gastric phase of digestion, cooked fat), I_CF (After intestinal phase of digestion, cooked fat), and similarly for the muscle tissue shown on the right. (B) Quantification of bta‐miR‐154c expression after cooking and digestion via qRT‐PCR. Experiments were repeated 5 times and are depicted as mean ± SD, **P < 0.01. Unpaired t‐tests were performed using graphpad Prism version 9.3.1 for Windows, GraphPad Software, La Jolla California USA. (C) Sequencing results after cloning of the digested bovine product into the pGEM‐T Vector (Promega, UK) and sequenced. The mature bta‐miR‐154c sequence was observed for all samples right after the vector sequence confirming that the mature bta‐miR‐154c molecules were still intact after all three phases of the *in vitro* digestion protocol. Negative control (blue colony with re‐ligated vector) does not contain the miRNA sequence. Sequences are followed by the adapter sequence for reverse transcription (A‐overhang).

The electrophoretic profile after each step of digestion is shown in Fig. 2A. RNAs up to 40 kDa size, corresponding to small RNA moieties, are found to be present after all three stages of digestion in both the adipose and muscle tissue samples. Subsequently, in order to specifically determine whether intact bta‐miR‐154c is included in the RNA repertoire present after all steps of digestion, quantitative real‐time PCR was performed using a bta‐miR‐154c‐specific primer. From a starting point of 30mg of tissue per sample, digestion resulted in an 87.5% (muscle) and 68.5% (adipose) concentration reduction in bta‐miR‐154c from equal amounts of raw undigested tissue. Still, bta‐miR‐154c was successfully detected after all phases of digestion (Fig. 2B) at a concentration of 0.43 ± 0.05 pm and 0.34 ± 0.05 pm (per 30mg of cooked/digested tissue) in the muscle and fat portion, respectively. The presence of mature bta‐miR‐154c sequence after all phases of digestion was further confirmed via sequencing analysis of the products as shown in Fig. 2C.

### Bta‐miR‐154c overexpressed in colorectal cell lines does not affect cell viability

**Fig. 3:**
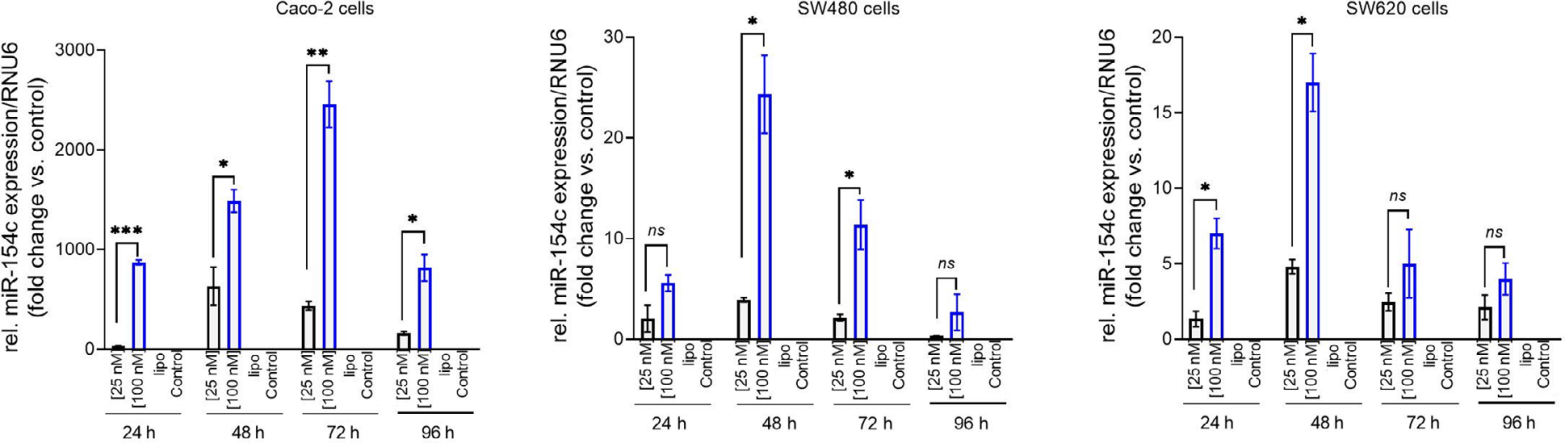
Determination of bta‐miR‐154c mimic expression levels at incubation times by qPCR after transient transfection of Caco‐2, SW480 and SW620 cells. Bta‐miR‐154c relative expression levels, compared with the RNU6, were determined by qPCR following transfection of the three cell lines with the mimic miRNA at a final concentration of either 25 nm or 100 nm. The three cell lines were also incubated with lipofectamine (lipo; negative control) or with the bta‐miR‐154c mimic without the use of transfection reagent (control). The bta‐miR‐154c mimic was detected in the cells at both the 25‐nm and 100‐nm concentrations, whereas it was not detected in either cell type incubated with lipofectamine or the mimic miRNA in the absence of the transfection reagent at all time points. Data are the means of three separate experiments normalized using RNU6 as an internal control and are presented as mean values ± SD. The student’s *t* test was employed to compare the miRNA expression levels after transfection of the cells with 25 nm or 100 nm of bta‐miR‐154c mimic at the different time points. Statistically significant differences are indicated with asterisks: *P < 0.05; **P < 0.01; ***P < 0.001, *ns*, not significant.

**Fig. 4:**
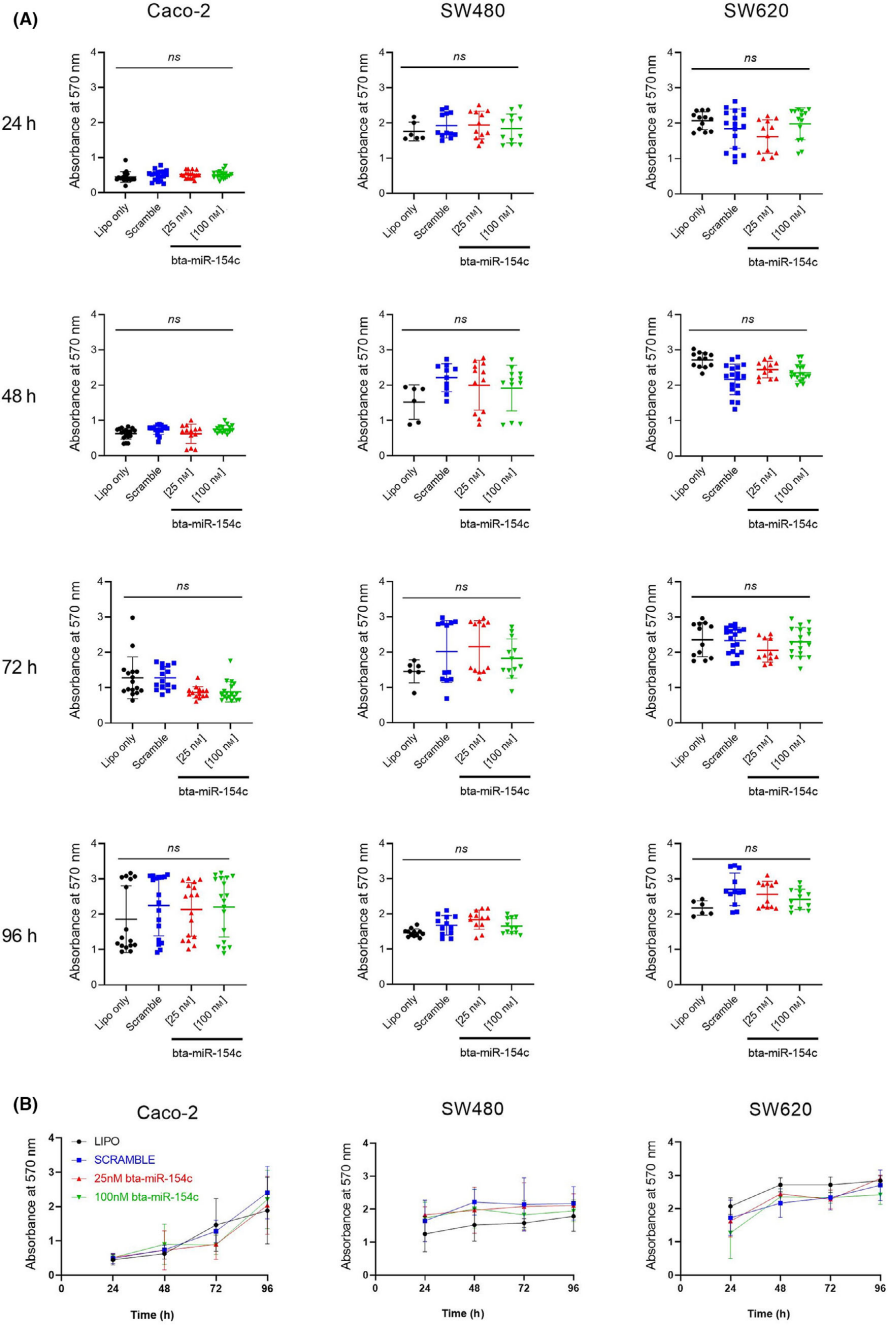
Bta‐miR‐154c overexpressed in colorectal cell lines does not affect cell viability. (A) MTT assay was performed for the determination of cell viability. Lipofection with bta‐miR‐154c at both a low [25 nm] and a high concentration [100 nm] had no effect on cell viability of any of the three cell lines tested, even when cells where challenged all the way to the 96‐h time point. Lipofectamine‐only treated cells and scramble sequence transfected cells on the high concentration were used as controls in these experiments. Data are the means of three separate experiments, with six measurements of OD in each experiment, and are depicted as mean ± SD. One‐way analysis of variance (ANOVA) was employed for comparisons between groups, ns—not significant. **(**B) Time course of the MTT assay. Time course to the 96‐h time point of the Caco‐2, SW480, and SW620 cells transfected with 25‐nm or 100‐nm bta‐miR‐154c mimic plus controls (lipofectamine‐only treated cells and cells treated with scramble sequence mimic) demonstrates that treated cells and controls are alive and dividing up to the 96‐h time point. Data are the means of three separate experiments, with six measurements of OD in each experiment and are depicted as mean ± SD.

Since bta‐miR‐154c was shown to withstand digestion, its functionality and bioactivity were then tested on human colon carcinoma cell lines. For this reason, we performed proof‐of‐principle experiments where bta‐miR‐154c mimic was transiently transfected at a low (25 nm) and a high (100 nm) concentration in three human CRC cell lines: Caco‐2, SW480, and SW620 cells. Cells were challenged at 4 different time points: 24, 48, 72, and 96 h. Transfection resulted in bta‐miR‐154c overexpression relative to the endogenous control (RNU6) in all cell lines tested both at the low (25 nm) and the high (100 nm) concentration throughout the 96‐h period as shown in Fig. 3. No bta‐miR‐154c expression was evident in cells treated only with lipofectamine, and in cells where bta‐miR‐154c (25 nm) was incubated without transfection reagent (RNA only control). After transfection, cell viability was determined using the MTT assay. For all cell lines treated either with 25‐nm or 100‐nm mimic, no significant difference in viability was evident as compared to the scrambled sequence transfected cells, or the lipofectamine‐only treated cells. This result was obvious for all time points tested all the way up to the 96‐h post‐treatment time point (Fig. 4).

## Discussion

The well‐established link between red meat consumption and CRC incidence [25], the recent data on the stability of miRNAs during meat processing [26, 27] and the ability of some miRNAs for cross‐kingdom regulation in the consumer [28], prompted us to examine the putative effects of bovine miRNAs on colorectal cancer progression.

Bovine miRNAs in edible parts of the animal were identified that do not share seed sequences with human miRNAs in order to be able to confidently identify and quantify them via qRT‐PCR and direct sequencing. We selected a bovine microRNA, miR‐154c, highly enriched in beef backfat that is predicted bioinformatically to affect many cancer‐related pathways should it reached human cells. The seed region of this miRNA is not conserved between bovine and humans. It is also absent in fish and poultry; however, the same sequence can be found in other red meats such as goat and sheep. When tested, bta‐miR‐154c was shown to be expressed both in adipose and muscle bovine tissue and to withstand both cooking and an *in vitro* protocol of human digestion albeit in reduced amounts. However, this miRNA did not alter cell viability, when the bta‐miR‐154c mimic was successfully transfected in three different intestinal cell lines.

Our data agree with previous studies depicting that edible, bovine tissues contain miRNAs that survive heat‐based preparation methods [26] and that bovine miRNA concentration when tested before and after roasting/cooking remains stable despite food processing [27].

MicroRNA stability throughout digestion is an active field of research, although, to our knowledge, studies are mostly targeted to plant miRNAs or miRNAs in bovine milk with contradicting results [29]. Some studies claim reduced miRNA withstand to digestion. Title et al., for example, found that less than 10% of mouse milk miR‐375 persisted after 2 h of incubation in intestinal fluid indicating degradation [30]. Also, Yang et al. showed that only a very small percentage of MIR2911 in cabbage extract was detected after an *in vitro* digestion protocol [31]. Similar results were reported by Huang et al, where corn miRNAs present either in AIN‐93M mouse diet or in extracts were extensively degraded through the *in vitro* digestion system used in the study [32]. High degradation was also observed in a study using artichoke miRNAs through a protocol of *in vitro* digestion [33]. On the other hand, various other studies have shown excellent stability of both plant [34, 35] and animal miRNAs through various *in vitro* digestion protocols [19, 32, 36, 37]. In our study, there was an 87.5% reduction in bta‐miR‐154c in muscle tissue after digestion, albeit the miRNA was still detected via qPCR and sequencing. These data show that there is a variability concerning both plant and animal miRNA stability when passed through *in vitro* digestion systems that requires further research. The discrepancies reported could be due to (a) different *in vitro* digestion conditions used, (b) different methods of detection, (c) the innate structure of the miRNA under study such as its sequence, structure, putative RNase A substrate motifs and GC content [31] and (d) its presence in extracellular vesicles, or bound to proteins, acting as protection agents. Indeed, extracellular vesicles such as exosomes have been shown to carry a cargo abundant in microRNAs increasing their stability by protecting them from RNase degradation [38, 39] Further experiments are required to shed light on this discrepancy. However, since, miRNAs could pose an effect on human mRNAs even at very small quantities [40], we tested whether this miRNA could still elicit a biological effect in human enterocytes.

In this study, we did not examine the natural absorption of the miRNA of interest into cells, but we performed proof‐of‐principle experiments by forcing via lipofection bta‐miR‐154c mimic into three different enterocyte cell lines and performing a cell viability assay. This is because incubation of bta‐miR‐154c without lipofectamine (RNA exposure condition), mimicking better the *in vivo* exposure scenario did not result in the detection of the microRNA mimic inside the cells (Fig. 3). For the transfected cells, no effect was obvious in the cells’ viability rates after transfection, at the various time points tested and after using two different concentrations (25 nm and 100 nm) of miRNA mimic (Fig. 4). Of course, our study was limited by the fact that only cell viability was tested using an intracellular metabolic assay such as MTT. Therefore, in future studies, we plan to examine the possible effects of bta‐miR‐154c, and other bovine miRNAs identified in this study, employing additional functional studies.

To our knowledge, the link between edible bovine miRNAs and CRC progression has not been addressed before. In a feeding study aimed to verify whether miRNAs from cooked beef were able to reach the human bloodstream, researchers concluded that a normal beef diet does not result in the horizontal delivery of miRNAs, but the study focused on miRNAs that have shared sequences with human miRNAs; therefore, direct identification was not possible [20]. In the case of CRC, though, ingested miRNAs do not need to necessarily cross the intestinal barrier and enter the bloodstream in order to exert their effect. They need to enter the enterocyte through its brush border membrane but not necessarily cross the basolateral membrane of the cell. In our study, we were able to detect and measure bta‐miR‐154c directly via qPCR and sequencing after all stages of digestion, meaning that this miRNA has the capacity to reach the human enterocytes upon ingestion. However, our study showed that overexpression of this miRNA mimic into the cells does not affect cell viability.

Our data agree with other studies where cross‐kingdom bioinformatic analysis did reveal dietary miRNA sequences with putative binding sites in human genes with carcinogen effects, but when evaluated *in vitro,* there was no proof of effect on cancer growth [15].

To conclude, our study showed that bta‐miR‐154 is predicted to have a tumorigenic effect in humans but found no evidence that it affects cell viability on the three colorectal cell lines tested here, even when overexpressed at supraphysiological concentrations. Of course, other pathways could be affected that have not been tested here, such as cell migration and proliferation. We also have to acknowledge that miRNA effects on specific mRNAs are concentration‐dependent meaning that the concentration of a miRNA may dictate, which genes it regulates, and overexpression could be masking any effects on cell viability [41, 42]. Also, we should note that we have not quantitatively analyzed the level of the bta‐miR‐154c in the 3 cell lines tested. It is known that miRNA concentrations in the cells are relevant to their function [43]. Notably, dietary miRNAs present in exosomes can have additional protective effects (not evaluated here) as previously shown in *in vitro* experiments for other miRNAs within similar cell lines [35]. Another limitation is that the effect observed in the current study is specific for miRNA bta‐miR‐154c and cannot be generalized to other microRNAs. One possibility of the lack of activity, despite the increased levels of the exogenous miRNA reached within the cells, is the reduced or null expression of some of the target genes of the studied miRNA. Indeed, in this study, we did not evaluate whether predicted target genes are indeed expressed in the cell lines tested. However, our study provides evidence that bta‐miR‐154c can still be detected after all stages of digestion albeit in reduced amounts. These data suggest that at least some bovine dietary miRNAs have the potential to reach the human intestinal lumen upon ingestion, and this result should not be overlooked. Since the gut is full of bacterial populations, further studies could shed light on the possible three‐way interaction between dietary miRNAs from red meat, the enterocytes, and the microbiota in the human gut. In the past, both endogenous [44] and dietary miRNAs [45] were shown to be implicated in such interactions with carcinogenic effects [46]. Further studies will shed more light on the effects described here.

## Conflicts of interest

All authors declare they have no conflicts of interests.

## Author contributions

MP and KF conceived and designed the project. HP, MF, FK, MAM, and GP acquired the data. MP, HD, GP, CP, and KF analyzed and interpreted the data. MP and KF wrote the paper. All authors read and approved the final manuscript.

## Supporting information


**Table S1**. Human genes predicted via cross‐species bioinformatic analysis to be affected by bta‐miR‐154c.

**Table S2**. Cancer‐related pathways in humans predicted via cross‐species bioinformatic analysis to be affected by bta‐miR‐154c.

**Table S3**. Diseases in humans predicted via cross‐species bioinformatic analysis to be affected by bta‐miR‐154c.

## Data Availability

The data that support the findings of this study are available in Tables 1 and 2 and the supplementary material of this article. Any further correspondence and requests for materials should be addressed to MP (pieri.m@unic.ac.cy).
